# Naive songbirds show seasonally appropriate spring orientation in the laboratory despite having never completed first migration

**DOI:** 10.1098/rsbl.2022.0478

**Published:** 2023-02-22

**Authors:** Joe Wynn, Bo Leberecht, Miriam Liedvogel, Lars Burnus, Raisa Chetverikova, Sara Döge, Thiemo Karwinkel, Dmitry Kobylkov, Jingjing Xu, Henrik Mouritsen

**Affiliations:** ^1^ Institut für Vogelforschung “Vogelwarte Helgoland”, An Der Vogelwarte 21, 26386, Wilhelmshaven, Germany; ^2^ AG ‘Neurosensorik/Animal Navigation’, Carl-von-Ossietzky Universität Oldenburg, 26111 Oldenburg, Germany; ^3^ Research Centre for Neurosensory Sciences, University of Oldenburg, 26111 Oldenburg, Germany; ^4^ MPRG Behavioural Genomics, Max Planck Institute for Evolutionary Biology, 24306 Plön, Germany; ^5^ Center for Mind/Brain Science, University of Trento, Piazza Manifattura 1, 38068 Rovereto, TN, Italy

**Keywords:** navigation, migration, orientation, inheritance, songbird, learning

## Abstract

The role of inherited orientation programmes in determining the outbound migratory routes of birds is increasingly well understood, though less is known about the influence of inherited information on return migration. Previous studies suggest that spatial gradient cues learnt through experience could be of considerable importance when relocating the natal site, though such cues could, in principle, augment rather than replace inherited migratory information. Here, we show that juvenile Eurasian blackcaps (*Sylvia atricapilla*) that have never left northwest Europe (i.e. never had the opportunity to learn navigational information on a continental scale) show significant spring orientation in a direction near-identical to that expected based on ringing recoveries from free-flying individuals. We suggest that this is probably indicative of birds inheriting an orientation programme for spring as well as autumn migration and speculate that, as long as the birds are not displaced far from their normal migration route, the use of inherited spring migratory trajectories might make uni-coordinate ‘stop signs’ sufficiently accurate for the long-distance targeting of their breeding sites.

## 1. Introduction

How birds precisely target their breeding sites (philopatry), often at great distances after months or even years have elapsed, is both remarkable and unsolved [1]. On outbound migration birds are typically thought to use an inherited migratory ‘clock and compass’ vector, though given the specificity with which some birds return to their breeding site it seems unlikely that birds rely solely upon a similar mechanism for return migration [2–8]. Spatially specific cues, learnt prior to departure (possibly via an imprinting mechanism) and uniquely associated with the breeding site, seem prime candidates when considering natal philopatry [1,5,9,10]. More specifically, cues varying systematically along a spatial gradient may offer a putative mechanism whereby birds can pinpoint the location of the natal site relative to their current position [11,12], and indeed multiple cues may be used together to form some type of bi-coordinate map [2,13]. Such a map could in principle allow birds to estimate the distance and direction to the goal, possibly even from beyond their known range [4,7,8,14]. Typically, the leading candidates for the sensory basis of an avian ‘map’ sense are considered to be the magnetic sense, or olfaction (for reviews, see [5,15]).

While learnt information is clearly important in long-distance migration, it is not entirely apparent whether such information overrides or augments inherited information. While a highly resolute positioning system would negate any requirement for an inherited programme, birds could in principle use learnt landmarks and spatial gradients to refine a course loosely determined by genetically inherited information [3,4]. In turn, this would reduce any reliance on the resolution at which learnt spatial information is remembered, which could be useful given that inter-annual variations in positions associated with spatial gradient cues can be substantial [16,17].

If birds were to use inherited information on their return migration, it would follow that they would take a seasonally appropriate migratory bearing in the absence of learnt cues even if they had never completed first migration. Here, we tested whether this was the case in Eurasian blackcaps (*Sylvia atricapilla*; ‘blackcaps’), a songbird with variable migratory distances (ranging from short distance to trans-Saharan) breeding across Europe. We investigated (i) whether naive birds (birds that have never left Northern Europe) assayed in a highly controlled laboratory environment showed significant orientation in the spring, and (ii) whether this orientation was consistent with the orientation of free-flying birds from a similar area (as assessed using ringing recoveries).

## 2. Methods

### (a) Experimental procedures

Birds were wild-caught in the vicinity of the University of Oldenburg (8.16^o^ E, 53.151^o^ N), Lower Saxony, across the years 2017 to 2022. Birds were included in this study if, based on plumage variation [18], they were aged as a juvenile (EURING age code ‘3’) and caught between 1 August and 1 September. We used 1 September as the cut-off threshold since the capture frequency of blackcaps on the nearby Vogelwarte Helgoland (Helgoland Bird Observatory), where blackcaps do not breed, was negligibly small in August and substantially higher in September [19]. This would imply that migratory blackcaps originating from Northern Europe were highly unlikely to be present in Northern Germany in August, hence by using birds captured before 1 September, we reasoned that the birds included in the study were unlikely to have started migrating at the point of capture and were likely nearby their breeding sites.

Birds tested in Emlen funnel experiments were kept indoors on-site under a light regime imitating the local photoperiod at a stable temperature (19.9°C). The birds were housed in pairs (if possible) in cages (1.05 m × 0.4 m × 0.4 m) during migratory seasons and overwintered in an indoor aviary, with all birds having *ad libitum* access to water and food (mealworms and desiccated insect protein). Birds were tested in Emlen funnels over the months of March–May in the respective years in indoor test chambers shielded from anthropogenic electromagnetic interference under normal magnetic field conditions (as described in [20,21]).

Because Emlen funnel data are inherently noisy, and the Rayleigh test of uniformity used for statistical analysis (see below) is necessarily weak (given it does not allow for multiple datapoints per individual; [22]), we sought to selectively remove inactive birds with poor migratory motivation in the funnel (inactive or random oriented escape behaviour). This is standard practice in such analyses (e.g. [20,21,23,24]). Importantly, we did not select birds based on orientation preference, meaning that birds were retained if they oriented repeatedly in *any* direction irrespective of what it was. To do this, birds were retained for analysis if they had been tested at least three times, were active within the Emlen funnel (greater than 30 scratches recorded on the funnel interior) on at least three occasions and showed an individual Rayleigh value of greater than 0.2 (see [23]). Of the birds for which we had a sufficient sample size, we retained 24 blackcaps (out of a total 52) for orientation analysis based on these criteria.

### (b) Estimation of expected migratory direction

To estimate an expected migratory direction for blackcaps in Northern Germany, we used ringing records of bird first caught in the breeding period within 250 km of the experimental site in Oldenburg and were subsequently recovered greater than 500 km away at the wintering site (*n* = 25; data obtained from the EURING). As above, we used capture rates on the nearby Helgoland Bird Observatory to define the breeding period, hence retaining in our analysis only birds caught between 1 May–31 August. A reanalysis using all birds captured within 250 km of Oldenburg is included in the electronic supplementary material, figure S1.

**Figure 1.**
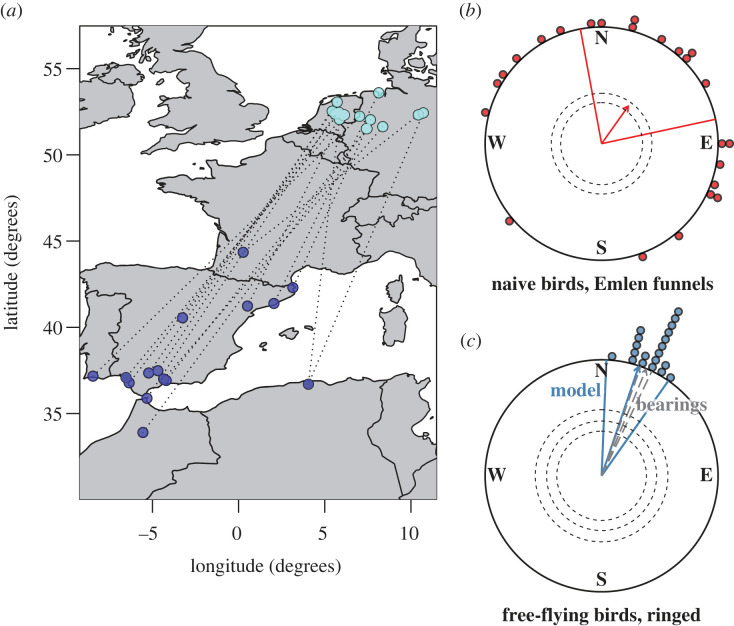
The orientation of naive songbirds compared to free-flying conspecifics. (*a*) A map showing the wintering locations (dark blue) and capture locations (light blue) of birds caught within 250 km of Oldenburg, with each set of records linked via a dotted line. (*b*) The spring orientation of naive birds tested in Emlen funnels, with a circular mean direction (± 95% CI) calculated from a mean bearing per bird and (*c*) the expected orientation of the birds shown in the left-hand map. Mean expected free-flying birds were calculated either as the circular mean of the observed bearings, or by using the inverse tangent of the change in latitude regressed against change in longitude.

We estimated an average migratory direction via two methods. First, for each ringing recovery, we calculated the loxodromic bearing (constant compass course) of movement between the wintering and breeding sites. From this, we calculated a circular mean ± 95% circular confidence intervals derived from a circular standard deviation [22]. Second, we calculated the inverse tangent of the gradient of the least means square linear regression of changes in longitude between ringing and recovery and changes in latitude between ringing and recovery (figure 1), from which we bootstrapped to obtain 95% confidence intervals about the estimated mean bearing. This allowed us to quantify the extent to which the orientation of free-flying birds overlapped that of the naive birds tested in Emlen funnels.

### (c) Statistical analysis

When considering the Emlen funnel orientation of blackcaps, we wanted to quantify two aspects: whether birds were oriented significantly, and if so whether their mean average orientation was in the *a priori* expected direction. To test for an overall significant preference in orientation direction, we used a Rayleigh test of uniformity. A mean direction was taken per bird from all orientation assays, and consistency was tested for within the distribution of the cohort overall (see [23]).

To test whether the observed orientation was in the *a priori* expected direction, we calculated a second-order circular mean for all birds tested in Emlen funnels. Additionally, we calculated bootstrapped 95% confidence intervals about the mean, sampling points with replacement from the overall sample and repeatedly calculating a circular mean direction. We assessed differences in direction between free-flying birds and naive birds tested in Emlen funnels by (i) ascertaining whether there was overlap in the confidence intervals of free-flying birds and naive birds, and (ii) assessing the extent of this overlap by comparing how closely the mean orientation of free-flying birds matched that of those in Emlen funnels. This, we reasoned, would allow us to assess whether naive birds differed significantly to free-flying birds.

## 3. Results

Using a Rayleigh test of uniformity, we found that naive birds were highly oriented when tested for orientational preferences in an Emlen funnel (*r* = 0.395, *p* = 0.022). Naive birds oriented with a mean direction of 35.4° (circular 95% CI: 352°, 80.4°) in Emlen funnels. Our estimate of likely migratory direction based on ringing recoveries was 18.6° when estimated using the inverse tangent of a linear model (bootstrapped 95% CI: 2.36°, 35.0°) and 22.6° (circular 95% CI: 19.6°, 25.6°) when calculated as the circular mean of the bearing between wintering and breeding sites.

## 4. Discussion

Despite having been caught as juveniles in northwest Europe, and hence having likely never experienced outbound migration, the mean directional preference exhibited by our naive Eurasian blackcaps was remarkably close to both that expected given the movements of ringed, free-flying birds and those tracked previously using geolocators [25]. This, we propose, is consistent with an inherited spring migratory direction that is independent of any learnt, spatial information. The juvenile birds considered in our analysis were not caught in the nest (as in other studies of orientation among naive migrants; e.g. [26]) and it is, therefore, possible that our result is driven by the limited amount of experience was gained before capture. However, we believe this is the less likely explanation of our results since (i) given the timing and location of capture it seems highly unlikely that our study birds had already started migration [19], and (ii) if birds were using learnt spatial information to determine orientation direction, we would not necessarily expect them to orient at all during the spring (given they were already at their breeding site). As such, we believe that innate orientation in a genetically inherited spring migratory direction seems the most parsimonious explanation.

Given the considerable and growing body of evidence for the use of learnt cues on return migration (e.g. [2,27–33]), and the remarkable precision with which some birds return to the natal site [34], it seems extremely unlikely that an inherited orientation vector is used in isolation on return migration in freely migrating birds. Nonetheless, considering the present results, it seems likely that learnt cues complement rather than replace those inherited, with innate orientation preferences therefore playing a surprisingly important role in return migration. This might make sense, especially given that philopatry, has been observed to be of lower precision in the offspring of parents that have emigrated into (rather than being born at) a given site [35]. In turn, this might imply that the probability of successful return has a heritable component that matches the breeding site, indicating a role for inherited information in returning to the breeding location.

If this were true, it would follow that the learnt information used to pinpoint the breeding location need not be particularly precise. Indeed, it has been postulated that a single dimension of spatial information (e.g. a latitudinal cue without a longitudinal counterpart) would be adequate for philopatry, with magnetic inclination specifically postulated as a possible candidate for such a uni-coordinate ‘stop sign’ mechanism [3,4,11]. The use of uni-coordinate information, magnetic or otherwise, is substantially less susceptible to year-on-year (secular) variation in the position denoted by cue values, owing to the potentially multiplicative effects of secular variation in multiple cues [16,17]. However, such a mechanism relies on the presence of an inherited vector to reduce ambiguity caused by multiple sites sharing the same cue value. Spring orientation of naive birds is, then, a key component of this proposed mechanism that we believe has thus far remained unobserved.

Irrespective of precisely how learnt information guides homewards orientation, our findings suggest that such learnt information augments rather than replaces an inherited spring orientational tendency. Not only does this suggest that the process of philopatry is perhaps more complex than has been previously thought, but also highlights the power of reanalysing existing Emlen funnel data to test novel hypotheses. We propose that, given the increasing abundance of similar orientation information, reanalysis could be a powerful tool when considering the holes remaining in our understanding of navigational ontogeny, and indeed in the study of animal behaviour more generally.

## Data Availability

The data are provided in the electronic supplementary material [36].
